# Overcoming gelling agent–derived inhibitory factors enables the cultivation of fastidious nitrifiers on solid media

**DOI:** 10.1093/ismeco/ycag246

**Published:** 2026-09-06

**Authors:** Zhiwei Peng, Atsushi Tamura, Tomonori Kindaichi, Akiyoshi Ohashi, Setsu Kato, Yutaka Nakashimada, Yoshiteru Aoi

**Affiliations:** Graduate School of Integrated Sciences for Life, Hiroshima University, Hiroshima 739-8530, Japan; Graduate School of Integrated Sciences for Life, Hiroshima University, Hiroshima 739-8530, Japan; Graduate School of Advanced Science and Engineering, Hiroshima University, Hiroshima 739-8527, Japan; Graduate School of Advanced Science and Engineering, Hiroshima University, Hiroshima 739-8527, Japan; Graduate School of Integrated Sciences for Life, Hiroshima University, Hiroshima 739-8530, Japan; Seto Inland Sea Carbon-neutral Research Center, Hiroshima University, Hiroshima 739-8511, Japan; Graduate School of Integrated Sciences for Life, Hiroshima University, Hiroshima 739-8530, Japan; Seto Inland Sea Carbon-neutral Research Center, Hiroshima University, Hiroshima 739-8511, Japan; Graduate School of Integrated Sciences for Life, Hiroshima University, Hiroshima 739-8530, Japan; Seto Inland Sea Carbon-neutral Research Center, Hiroshima University, Hiroshima 739-8511, Japan

**Keywords:** nitrifying microorganisms, microbial cultivation, uncultured microbes, non-colony-forming, gelling agents, solid medium, toxicity

## Abstract

Nitrification is a key process in the global nitrogen cycle, yet the physiological characterization of nitrifiers remains limited due to their low culturability. Although culture-independent approaches have provided important ecological insights, they do not fully resolve the physiological properties of nitrifying microorganisms. Here, we show that common gelling agents (agar, agarose, and gellan gum) contain toxic substances that inhibit the growth of diverse nitrifying microorganisms as well as some heterotrophic bacteria. These inhibitory substances appear to be generated during autoclaving and act via mechanisms distinct from previously reported hydrogen peroxide. Removal of these inhibitory factors using a simple washing strategy enables colony formation in recalcitrant nitrifiers, including *Nitrososphaera* (ammonia-oxidizing archaea) and *Nitrospira* (nitrite-oxidizing bacteria). Application of this approach to environmental samples enabled the isolation of multiple difficult-to-culture nitrifying strains, including ammonia-oxidizing and nitrite-oxidizing microorganisms. Together, these findings demonstrate that the limited culturability of nitrifiers on solid medium is largely driven by gelling agent**–**derived inhibitory substances and can be overcome by a simple and broadly applicable strategy.

## Introduction

Nitrification is a critical process in biogeochemical nitrogen cycle. It profoundly impacts wastewater treatment, agricultural fertilization efficiency, and greenhouse gas nitrous oxide emissions. Consequently, gaining a deep physiological understanding of nitrifiers is essential [1, 2].

Culture-independent methods currently dominate the study of the ecology and ecophysiology of nitrifiers. Molecular approaches, including 16S ribosomal RNA (rRNA) gene–based and metagenomic analyses, provide phylogenetically informed descriptions of microbial community structure, and also enable partial linkage of taxonomic identity with functional potential, even for uncultivated microorganisms. Together, these approaches provide valuable insights into the ecophysiological characteristics of nitrifying microorganisms [3–6]. However, strong reliance on culture-independent methods limits our fundamental physiological understanding of nitrifiers. Therefore, obtaining pure cultures remains irreplaceable for detailed and rigorous physiological characterization. For example, pure cultures enable the direct determination of key traits such as microbial kinetics [7–9]. Moreover, pure culture-based studies are essential for validating metabolic capabilities, assessing responses to specific conditions, identifying regulatory and interaction mechanisms, and discovering unexpected novel functions [10–13].

Obtaining pure cultures under laboratory conditions remains a formidable challenge. At present, most microorganisms remain uncultivated [14–17]. The cultivation of nitrifiers faces a similar bottleneck, with <6% of nitrifying species having cultured representatives (Table S1). It is also widely recognized that nitrifiers are inherently difficult to isolate and cultivate due to their relatively slow growth rates. Therefore, how to overcome these barriers and then bring more nitrifiers into culture remains an important theme in bioecology and biogeochemistry.

At present, microbial cultivation relies on two distinct approaches: solid medium cultivation methods (e.g. agar plating) and liquid medium cultivation methods (e.g. dilution-to-extinction). Solid medium serves as the primary method used for microbial cultivation, offering superior advantages over liquid methods. Solid medium often provides higher throughput capacity than liquid methods; enables visual discrimination of diverse microbial groups through colony phenotypes; and allows straightforward colony picking, clonal purification, and maintenance of microbial isolates.

Liquid culture-based methods, including dilution-to-extinction cultivation, have nevertheless been widely used for the isolation of nitrifiers. However, because nitrifiers typically attain relatively low cell densities and do not produce visible turbidity, growth is often difficult to assess by visual inspection alone. Consequently, growth frequently must be confirmed by microscopic observation, Polymerase Chain Reaction (PCR)-based detection, or chemical analyses of substrate consumption and product formation. Furthermore, positive cultures may still contain mixtures of nitrifiers and accompanying heterotrophic bacteria, requiring repeated transfers and verification before pure cultures can be obtained. In contrast, colony formation on solid media enables direct growth detection, colony picking, and clonal purification, substantially improving the efficiency of obtaining and maintaining pure nitrifier isolates.

Applying solid medium to cultivate nitrifiers presents a major challenge; it is widely recognized that most nitrifiers cannot form colonies on solid medium. The original isolates of most nitrifying species were obtained by liquid methods; specifically, none of the original isolates of ammonia-oxidizing archaeon (AOA) or nitrite-oxidizing bacterium (NOB) species were obtained by solid medium (Table S2) [11, 12, 18–56].

The isolation of nitrifiers still commonly relies on liquid culture-based methods. For the enrichment of target microbes before inoculation into liquid media, various novel technologies such as cell sorter-based method and high-throughput microfluidics have been developed [12, 18, 55, 57]. Although these methods have indeed brought some new nitrifiers into culture, they mainly improve the upstream enrichment or separation of target microbes from complex mixtures and do not resolve the downstream challenges associated with growth verification, and maintenance of isolates in liquid cultivation systems. Therefore, identifying the factors that prevent colony formation on solid media is essential for improving the efficiency of nitrifier isolation.

In this study, we demonstrate that growth inhibition on solid medium is largely driven by toxic substances present in commonly used gelling agents. We then validate the effect of removing these inhibitory factors using representative nitrifiers, including recalcitrant lineages, such as *Nitrososphaera* and *Nitrospira*, which are typically unable to form colonies. Finally, we apply this approach to environmental samples and demonstrate its practical utility by successfully isolating previously uncultivated or difficult-to-culture nitrifying microorganisms. This approach provides a practical and broadly applicable solution to improve the culturability of environmental microorganisms. We further show that this inhibitory effect extends beyond nitrifiers to some heterotrophic bacteria, suggesting a more general constraint on solid-medium cultivation. These findings suggest that the limited culturability of nitrifiers on solid medium is not solely an intrinsic property of nitrifiers, but is strongly influenced by inhibitory effects associated with gelling agents.

## Materials and methods

### Bacterial strains and media compositions

All pure cultures employed in this study are listed in Table S3.

*Nitrospira* sp. ND1 [55], *Nitrospira japonica* J1 [18], *Nitrospira* sp. KM1 [58], strain No.1 [59], strain No.120 [60], strain No.154 [60], and strain No.206 [60] were isolated in previous research; these strains are continuously maintaining in our laboratory. *Nitrobacter* sp. strain N26, *Nitrosomonas* sp. strain A9, and *Nitrosomonas* sp. strain A15 were isolated in this research using the microcolony-targeted isolation method as described in our previous study [18, 55]. *Nitrososphaera viennensis* EN76 and *Nitrosopumilus adriaticus* NF5 were obtained from the Japan Collection of Microorganisms (JCM, RIKEN BioResource Research Center, Tsukuba, Japan). Strains No.62 and No.508 were isolated by 10% R2A agar medium in this research.

NOB strains were cultivated using NOB-medium containing 98.6 mg L^−1^ NaNO₂, medium composition other than NaNO_2_ corresponded to our previous research [55]. The pH was adjusted to 7.5–7.8 before incubation.

*Nitrosomonas* strains were cultivated using ammonia-oxidizing bacteria (AOB)-medium containing 76 mg L^−1^ NH₄Cl, 34 mg L^−1^ KH₂PO₄, 116 mg L^−1^ NaCl, 40 mg L^−1^ MgSO₄·7H₂O, 73 mg L^−1^ CaCl₂·2H₂O, 38 mg L^−1^ KCl, 0.236 mg L^−1^ FeSO₄·7H₂O, 100 mg L^−1^ KHCO₃, 0.015 mg L^−1^ MnSO₄·5H₂O, 0.025 mg L^−1^ CuSO₄·5H₂O, 0.062 mg L^−1^ H₃BO₃, 0.056 mg L^−1^ ZnSO₄·5H₂O, 0.024 mg L^−1^ Na₂MoO₄·2H₂O, 0.029 mg L^−1^ Co(NO₃)₂, and 0.024 mg L^−1^ NiCl₂·6H₂O. The pH was adjusted to 7.8 before the medium was used, and the medium was buffered with 20 mM HEPES.

*Nitrososphaera viennensis* EN76 and *N. adriaticus* NF5 were cultivated in defined mineral media following previously published protocols [45, 61].

All heterotrophic strains employed in this study were cultivated in liquid medium which is composed of 10% R2A Broth (395-01681, Shiotani M.S. Co., Ltd).

All cultures were incubated statically in the dark at 28°C, except for *N. viennensis* EN76, which was incubated at 42°C.

### Environmental sample collection and enrichment cultivation

Three environmental samples from different habitats were collected to enrich nitrifiers.

The activated sludge sample was obtained in 2023 from a municipal wastewater treatment plant (100-1 Saijocho Taguchi, Higashihiroshima, Hiroshima 739-0036, Japan) for the enrichment of *Nitrospira*. The culture has been maintaining in a continuous-flow bioreactor for over 2 years, providing nitrite continuously according to the same manner described in our previous study [62].

Freshwater and soil samples were collected from the Hiroshima University campus in January 2025 for the subsequent enrichment of ammonia-oxidizing microorganisms (AOMs) and NOB by serial transfer. The freshwater sample associated with a biofilm on submerged rocks was collected from Budō Pond (34° 24′ 04.6″ N, 132° 42′ 45.6″ E), and the soil sample was collected from a depth of 5–10 cm in an agricultural field (34° 23′ 45.7″ N, 132° 43′ 02.2″ E). Each sample was inoculated into two sets of modified AOB-medium, where the NH₄Cl concentration was adjusted to either 20 mg-N L^−1^ or 120 mg-N L^−1^, and HEPES was replaced with 1 g L^−1^ of CaCO₃; other medium composition corresponded to that of the AOB-medium described above. Cultures were incubated at 24°C in the dark with gentle shaking. Upon complete consumption of the supplied ammonium (monitored using commercial ammonium test paper), 10% (v/v) of the culture was transferred into fresh medium. This cycle repeated for over 4 months after nitrifying activity reached a steady state.

### Preparation of solid-based culture media

Three types of solid-based culture media were employed in this study: conventional solid medium, the “Liquid–Solid Method” [63], and “washed solid medium.”

Conventional solid medium was prepared by supplementing the corresponding culture medium with 1.5% (w/v) agar (01028-85, NACALAI TESQUE, INC); 1.5% (w/v) agarose (312-01193, NIPPON GENE); or 0.8% (w/v) gellan gum (075-03075, FUJIFILM Wako Pure Chemical Corporation). Gellan gum medium was additionally supplemented with 0.025% (w/v) CaCl₂ to facilitate gelation.

Washed solid medium was prepared by washing conventional solid medium with liquid medium to remove potential inhibitors. Specifically, conventional solid medium was first prepared in either a 25 cm^2^ tissue culture flask (for cultivation of pure cultures) or a 125 ml jam jar (for isolation of environmental microorganisms). Then, liquid medium (twice the volume of conventional solid medium) was gently added to the vessel. The vessels were shaken overnight at 90 rpm, after which the liquid phase was discarded. This washing step was repeated twice to ensure thorough removal of soluble impurities.

**Figure 1:**
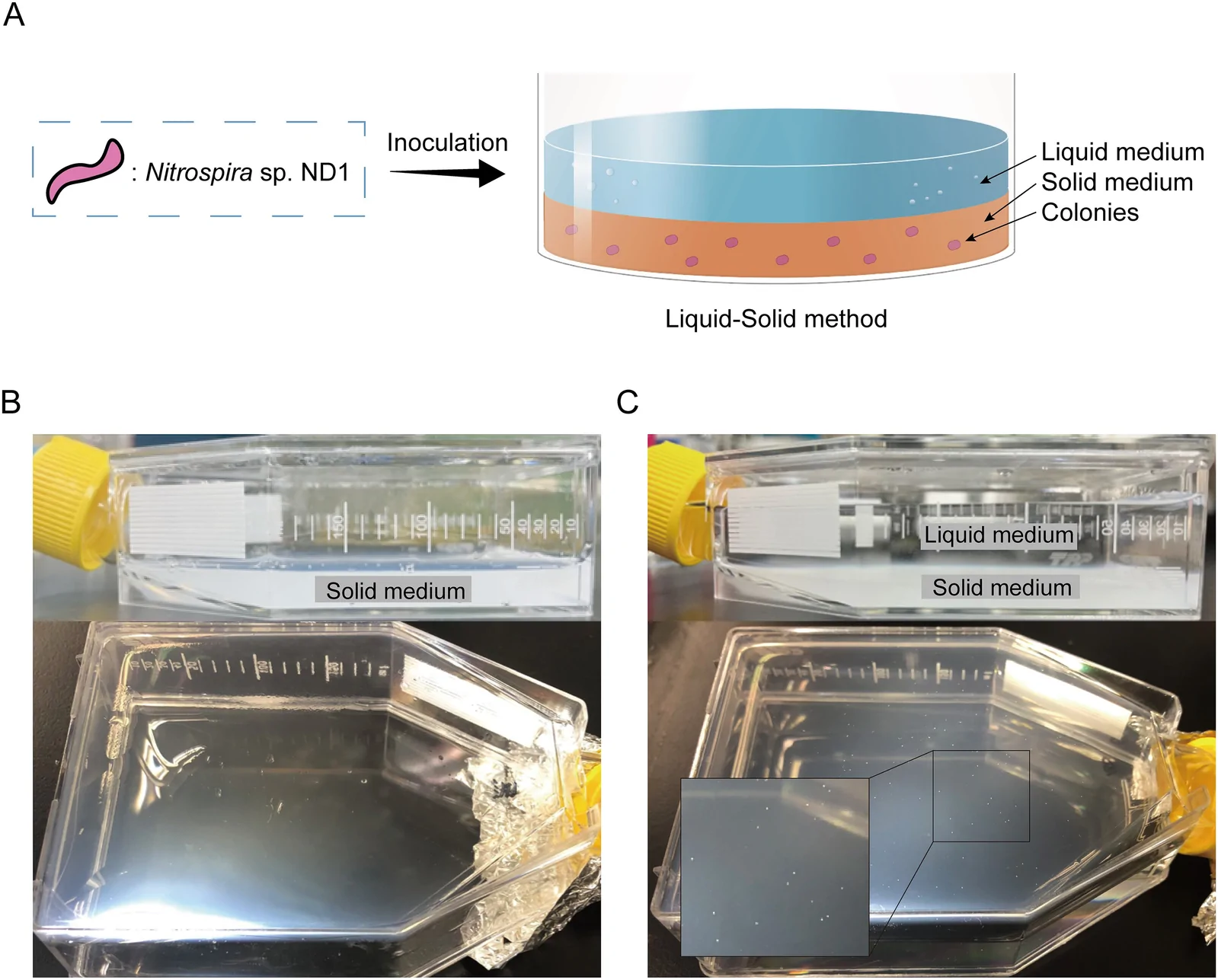
Comparison of colony formation by *Nitrospira* sp. ND1 on conventional solid medium versus the liquid–solid method. (A) Experimental conception of cultivation *Nitrospira* sp. ND1 using liquid–solid method. (B) Cultivation of cells using conventional solid medium (solidified by 1.5% agar). The top panel shows the side view of the flask containing only solid medium, while the bottom panel (top–down view) reveals the absence of visible colonies after incubation. (C) Cultivation of cells using the liquid–solid method. The top panel illustrates the setup where a liquid medium overlay is maintained above the solid agar base. The bottom panel demonstrates the formation of distinct colonies (highlighted in the magnified figure) under these conditions.

The liquid–solid method involves embedding cells in solid medium while maintaining contact with a liquid phase. Briefly, 10 ml of 1.5% agar medium was first prepared. After cooling to ~40°C, an actively growing bacterial culture was added to the molten agar and mixed thoroughly to achieve uniform cell distribution. The mixture was then poured into a 25 cm^2^ tissue culture flask. Once solidified, 10 ml of liquid culture medium was gently added on top of the agar surface (Fig. 1A). The liquid–solid setup was used solely as a preliminary, hypothesis-generating experiment to examine whether continuous contact with a liquid phase could alleviate growth inhibition and was not evaluated as an isolation method for comparison with washed solid medium.

All solid-based culture media were prepared by the Phosphate Separately(PS) method [64], and the autoclave was performed under 121°C for 15 min.

### Extracting the substances from gelling agents

**Figure 2:**
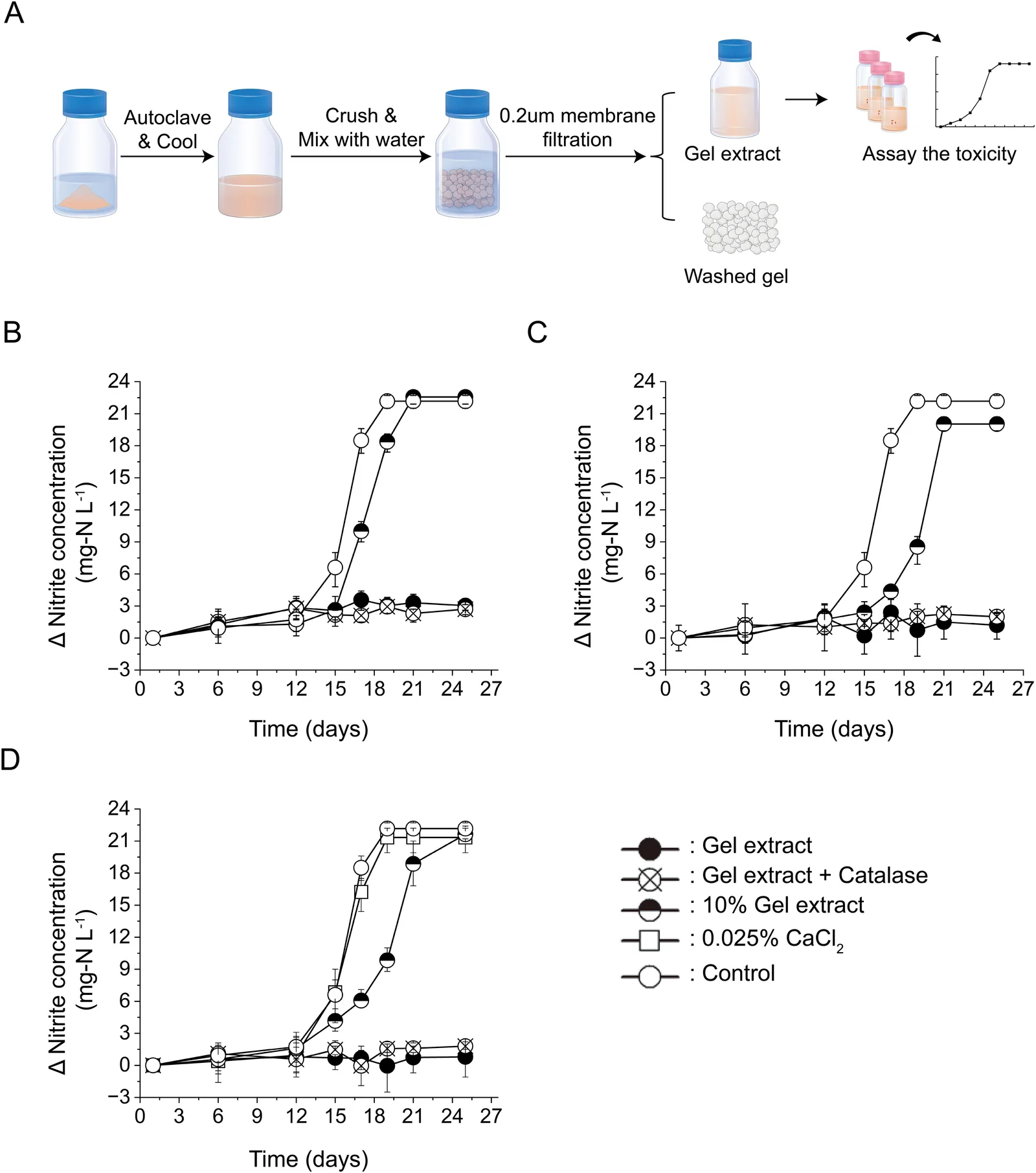
Effect of gel extracts on the growth of *Nitrospira* sp. ND1. (A) Experimental conception of extracting toxic substances from gelling agents and assaying the toxicity of gel extract. (B–D) Growth of *Nitrospira* sp. ND1 in liquid medium with extract of agar (B), agarose (C), or gellan gum (D). Gel extract was prepared by extracting autoclaved and crushed 6% agar, 6% agarose, or 3.2% gellan gum with DI in equal volume, then filtered by 0.2 μm pore size membrane. *X*-axis, incubation time (days); *Y*-axis, change in nitrite concentration (ΔNO₂^−^, mg-N L^−1^). Data represent means ± SD (*n* = 3).

The extraction procedure was described as follows: agar (6%, w/v), agarose (6%, w/v), or gellan gum (3.2%, w/v) powder was first dissolved in Deionized (DI) water and sterilized by autoclaving. These concentrations were selected to mimic the concentrations of gelling agent**–**derived compounds present in standard solid media (1.5% agar, 1.5% agarose, and 0.8% gellan gum) within the subsequent liquid bioassays. Because the extraction procedure and bioassay (see below) each involved a two-fold dilution step, four-fold higher gel concentrations were used during extraction preparation. After solidification, the gel was crushed into <1 cm^3^ size, and an equal volume of DI water was added. The mixture was shaken overnight at 90 rpm, and the liquid phase (referred to as the “gel extract”) was separated from the gel residue by 0.2 μm pore size membrane filtration. The gel residue was then processed through two additional washing cycles, each involving the addition of fresh liquid medium followed by the same shaking and filtration steps. The resulting solid matrix was referred to as “washed gel” (Fig. 2A). Extract of re-autoclaved washed gel (ERWG) was prepared by re-autoclaving the washed gel and repeating the extraction procedure.

### Cultivation of strains on solid-based culture media

All strains were inoculated onto conventional solid medium to evaluate their colony-forming ability; strains that did not form colonies on conventional solid medium were subsequently inoculated onto washed solid medium. Strain ND1 was additionally cultivated using the liquid–solid method (Table S3). Approximately 3000–5000 cells were inoculated onto each medium and incubated in the dark under static conditions for a maximum of 80 days. Colonies that formed on washed solid medium were subsequently transferred to liquid medium to assess their activity.

### Cultivation of strains with substances derived from gelling agents

All strains were cultivated in liquid medium supplemented with gel extract. Strain ND1 was additionally employed to evaluate the growth inhibitory effect of washed gels, ERWGs, and 0.025% CaCl_2_ (Table S3).

To assess whether H₂O₂ contributed to the observed growth inhibition, a catalase stock solution (2 mg ml^−1^, specific activity, 9700 U mg^−1^) was filter-sterilized and added to the gel extract assay mixtures at 1 ml L^−1^, yielding final concentrations of 2 mg L^−1^ and 19.4 U ml^−1^. Prior to catalase treatment, H₂O₂ concentrations in these mixtures were determined using the copper(II)-2,9-dimethyl-1,10-phenanthroline (DMP) spectrophotometric method with minor modifications [65]. Absorbance was measured at 492 nm using an H₂O₂ calibration curve, with an experimentally determined detection limit of 1 μm. To verify the efficacy of catalase under the assay conditions, parallel mixtures supplemented with 10 mM H₂O₂ were treated with the same catalase dosage for 10 min, and the residual H₂O₂ concentration was determined using the same method.

Each bioassay was conducted in a 50 ml Bayer bottle containing 10 ml of substances extracted from gelling agents, 8 ml of 2.25 times concentrated liquid culture medium, and 2 ml of actively growing cell suspension at ~10^6^ cells ml^−1^. The growth of nitrifiers was evaluated by nitrite concentration, and the nitrite concentration was measured by Griess reaction-based spectrophotometry as described in our previous study [55], and the growth of heterotrophs was evaluated by measuring optical density at 600 nm (OD_600_). Measurements were taken every 2–3 days until no further change was observed in the control group. Incubation was conducted in the dark at 28°C.

### Isolation of environmental nitrifiers using washed solid medium

Gellan gum-based washed solid medium was applied in the isolation of environmental nitrifiers. Enrichment cultures from activated sludge, freshwater, and soil were first passed through a 10 μm membrane filter to remove large particles and aggregates before inoculation onto washed solid medium.

For the activated sludge enrichment, NOB were isolated using washed solid medium prepared with three modified NOB-media containing different nitrite concentrations (5, 10, and 20 mg-N L^−1^), based on the NOB-medium described above. After 45 days of incubation, individual colonies were picked and transferred to liquid NOB-medium to confirm nitrite-oxidizing activity, and positive cultures were subsequently identified by 16S rRNA gene amplicon sequence.

For the soil and freshwater enrichments, nitrifiers were isolated using washed solid medium prepared with three different medium formulations: two modified AOB-media (matching the NH₄^+^ concentrations used during the respective batch enrichments) and one NOB-medium. After 45 days of incubation, colonies were manually picked into 96-well plates with corresponding liquid media to verify nitrifying activity, and active cultures were identified by high-throughput 16S rRNA gene amplicon sequencing.

For all washed solid medium plates, ~3000 cells were inoculated per plate, and incubations were carried out in the dark at 28°C.

### Identification of nitrifying activity positive culture by 16S rRNA gene sequencing

#### Cultures originated from activated sludge enrichment

16S rRNA gene of each culture was directly amplified via PCR amplification using the primer pair 27F-mod (5′–AGRGTTTGATYMTGGCTCAG–3′) and 1492R (5′–GGTTACCTTGTTACGACTT–3′) with KOD One PCR Master Mix (Toyobo Co., Ltd, Osaka, Japan). The resulting amplicons were purified and subjected to Sanger sequencing at Eurofins Genomics Co., Ltd (Tokyo, Japan), using the internal primers 515F (5′–GTGCCAGCMGCCGCGGTAA–3′) or/and 806R (5′–GGACTACHVGGGTWTCTAAT–3′). The obtained sequences were compared with the GenBank database (http://blast.ncbi.nih.gov/) to identify the closest matching sequences using BLASTn [66–68].

#### Cultures originated from soil and freshwater enrichment

The V4 region of the 16S rRNA gene was directly amplified from 180 nitrifying activity positive cultures via PCR amplification. The 180 cultures were divided into six independent groups, with 30 cultures per group. Within each group, amplification was performed using the primer pair 515F-mod (5′–Barcode + GTGYCAGCMGCCGCGGTAA–3′) and 806R-mod (5′–GGACTACNVGGGTWTCTAAT–3′) [69], where a set of 30 unique barcodes was incorporated into the forward primers to distinctly tag the 30 cultures [70, 71]. Amplicons within the same group were then pooled together in equal amounts of DNA, resulting in six distinct multiplexed samples. These six samples were submitted to Seibutsu Giken Inc. (Kanagawa, Japan) for high-throughput sequencing on an MiSeq System (Illumina).

For downstream data processing, the raw sequencing data generated from the six samples were processed. Reads within each of the six datasets were demultiplexed based on their internal barcodes to reassign the sequences to the original 180 individual cultures. The demultiplexed reads were subsequently quality-filtered and analyzed using the QIIME 2 microbiome bioinformatics platform (version 2025.7). Taxonomic classification of the sequences was performed using a classifier trained on the Greengenes database (version 13.8).

## Results and discussions

### Colony formation of pure cultures on conventional solid medium

The colony-forming ability of the employed strains was evaluated using conventional solid medium. In detail, eight nitrifiers and six heterotrophs (including three positive controls) that exhibited robust growth in liquid medium were inoculated onto conventional solid medium, which was solidified with 1.5% (w/v) agar, 1.5% (w/v) agarose, and 0.8% (w/v) gellan gum (Table S3).

The three positive controls grew well on conventional solid media after 45 days of incubation, but most tested nitrifying strains (Table 1) and other heterotrophs (Table S4) failed to form colonies, regardless of the gelling agent used.

**Table 1:** Growth responses of strains to conventional solid medium and gel extracts.

Functional group	Strain ID	Closest species	Similarity (%)	Growth on conventional solid medium solidified with			Growth in liquid medium with extract of		
				Agar	Agarose	Gellan gum	Agar	Agarose	Gellan gum
NOB	ND1	*N. defluvii*	99.8	−	−	−	−	−	−
	J1	*N. japonica*	100	−	−	−	−	−	+
	KM1	*N. japonica*	98	−	−	−	−	−	−
	N26	*N. vulgaris*	99.8	+	−	−	+	−	−
AOB	A9	*N. oligotropha*	97.6	−	−	−	−	−	−
	A15	*N. ureae*	95.6	−	−	−	+	−	+
AOA	EN76	*N. viennensis*	100	−	−	−	−	−	−
	NF5	*N. adriaticus*	100	−	−	−	−	−	−
Positive control	No.1	*Escherichia coli*	100	+	+	+	+	+	+
	No.62	*Nocardioides pyridinolyticus*	97	+	+	+	+	+	+
	No.508	*Skermanella aerolata*	92.39	+	+	+	+	+	+

The table summarizes the growth behavior of nitrifying strains and positive control strains employed in this study. “Closest species” and “Similarity (%)” refer to the nearest described species based on Sanger sequences. Colony-forming ability was tested on conventional solid medium solidified with agar, agarose, or gellan gum (“Growth on conventional solid medium solidified with…”). Growth inhibitory effect of gel extracts was tested in the corresponding basal medium supplemented with extract of agar, agarose, or gellan gum (“Growth in liquid medium with extract of…”). Symbols indicate growth responses: “+” denotes visible growth, whereas “–” indicates no detectable growth.

These results suggest that colony formation of these strains was inhibited by factors associated with growth on solid media. Such factors may include diffusion limitation, accumulation of metabolic by-products, microenvironmental effects associated with the solid matrix, and inhibitory compounds derived from gelling agents. Furthermore, the observed inhibition was not restricted to particular species but appeared to affect a broad range of the microorganisms tested. In the present study, we specifically focused on evaluating the potential contribution of gelling agent**–**derived inhibitory compounds to microbial growth.

### Growth of *Nitrospira* sp. ND1 using liquid–solid method

To investigate whether an overlaid liquid medium could mitigate the growth inhibition on conventional solid medium, *Nitrospira* sp. ND1, which failed to form colonies on solid medium in this study, was used as a model organism in a liquid–solid method. This method involved mixing bacterial cultures with molten conventional solid medium, followed by the addition of an equal volume of liquid medium as an overlay after solidification (Fig. 1A). As expected, *Nitrospira* sp. ND1 did not grow on the conventional solid medium (Fig. 1B) but formed colonies embedded within the agar matrix when using the liquid–solid method (Fig. 1C). Colony formation rates (Equation 1) reached ~9% after 60 days of incubation, which further increased to 12% by Day 74. These observations are consistent with the previous report of improved cultivation of AOA strains using a similar approach [63].


(1) \begin{eqnarray*} \mathrm{Colony}\ \mathrm{formation}\ \mathrm{rate}=\frac{\mathrm{Number}\ \mathrm{of}\ \mathrm{formed}\ \mathrm{colonies}}{\mathrm{Number}\ \mathrm{of}\ \mathrm{inoculated}\ \mathrm{cells}}\# \end{eqnarray*}

Given the critical role of the liquid phase in colony formation, we postulated that it may reduce growth inhibition by diluting water-soluble toxic substances within gelling agents, thereby creating a permissible environment for colony formation.

### Growth of *Nitrospira* sp. ND1 is inhibited by gel extract

To clarify whether water-soluble toxic substances from gelling agents were responsible for microbial growth inhibition on conventional solid medium, *Nitrospira* sp. ND1 was cultivated in liquid medium which was supplemented with extracts of agar, agarose, and gellan gum gel at different concentrations (Fig. 2A).

Extracts from all three tested gelling agents completely inhibited nitrite oxidation relative to controls. Although this inhibition was reduced at lower extract concentrations (10% v/v), nitrite oxidation remained compromised. To exclude the possibility that H₂O₂ contributed to the growth inhibition, we further measured its concentration in mixtures containing gel extracts and culture medium and found it to be below the detection limit of 1 μm. To further rule out any inhibitory contribution from trace H₂O₂ below the detection limit, catalase was added to remove any residual H₂O₂. In an independent validation experiment, the same catalase dosage reduced 10 mM H₂O₂ to below the detection limit within 10 min, confirming its effectiveness under the assay conditions. As a result, the addition of catalase to the culture did not restore growth, suggesting that hydrogen peroxide (H₂O₂) is unlikely to be the primary cause of the observed inhibition (Fig. 2B–D).

H₂O₂ has long been considered as a major factor causing the toxicity to microorganisms. Especially, the interaction between agar and phosphate buffer during autoclaving generates significant levels of H_2_O_2_, which severely inhibits the culturability of diverse environmental bacteria [64, 72, 73]. H_2_O_2_ was also demonstrated as a critical growth inhibitor for AOA [74]. Furthermore, trace metals in culture media can synergistically catalyze H₂O₂ formation under aerobic conditions, exerting profound toxicity on sensitive autotrophs [75]. In contrast, the observed toxicity in this study appears to be driven by a completely different, H₂O₂-independent mechanism, suggesting the presence of other as yet uncharacterized inhibitory substances derived from the gelling agents.

### Widespread inhibition of microbial growth caused by gel extracts

To determine whether the toxicity of gel extracts also affects the inability of other employed strains to form colonies on conventional solid medium, we further evaluated their growth inhibitory effects across these microbial taxa.

**Figure 3:**
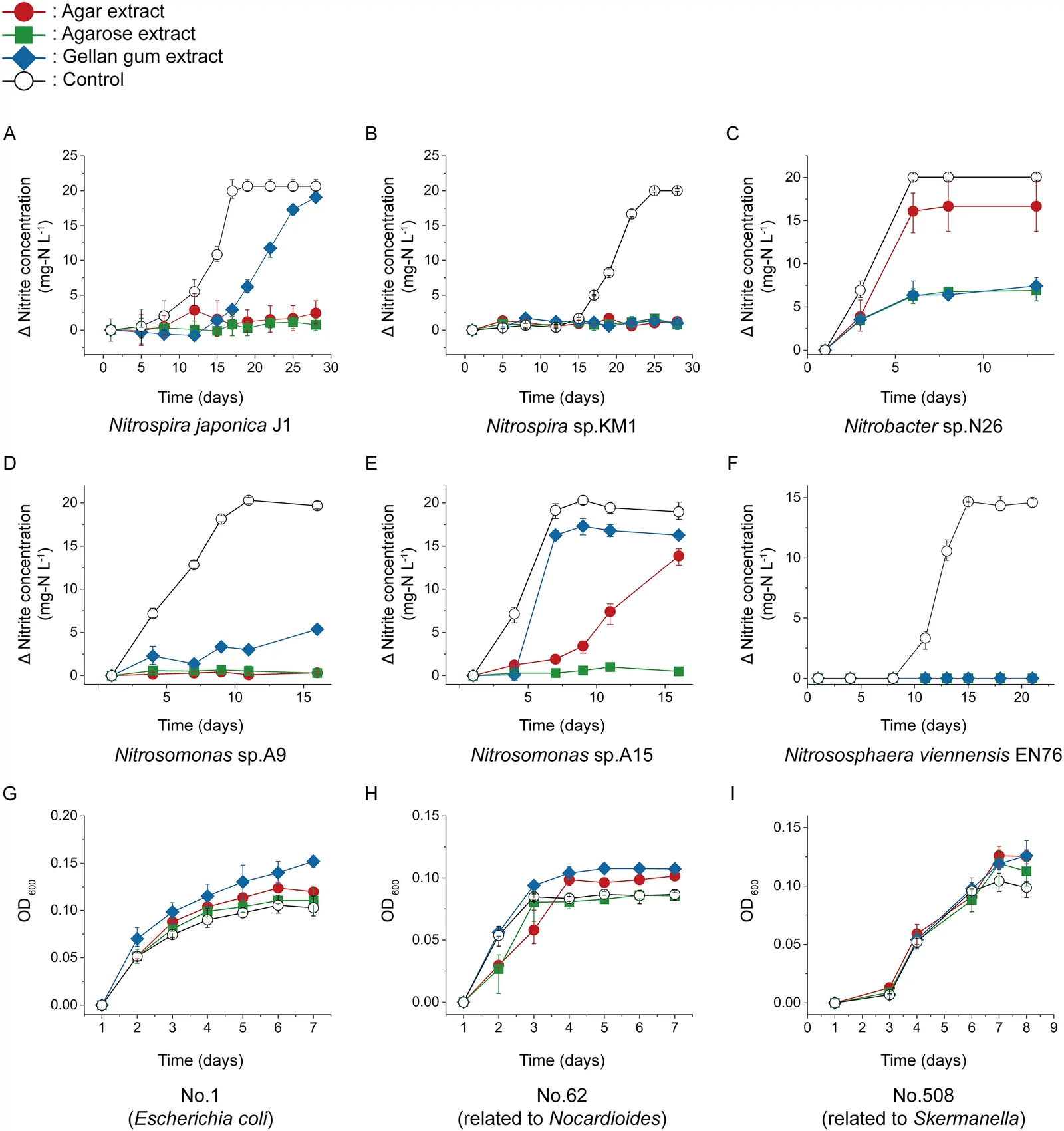
Effect of gel extract to the growth of other nitrifiers and positive controls. Growth of NOBs (A–C); AOMs (D–F) and positive controls (G–I) in liquid medium with extract of agar, agarose, or gellan gum. *X*-axis, time (days); *Y*-axis, the growth of strains, for NOB and AOM, growth was evaluated as the changes in nitrite concentration (ΔNO₂^−^, mg-N L^−1^), whereas for positive controls growth was evaluated as optical density at 600 nm (OD_600_). Data represent means ± SD (*n* = 3).

The growth inhibition patterns of nitrifiers in liquid culture with gel extract were largely consistent with colony-forming phenotypes on conventional solid medium: the growth of strains which failed in forming colonies on conventional solid medium was inhibited by the corresponding gel extracts (Fig. 3 and Table 1), and this similarly strong correlation was also observed for the tested heterotrophic strains (Fig. S1 and Table  S4).

Some exceptions were observed. For example, *N. japonica* J1 (Fig. 3A) and *Nitrosomonas* sp. strain A15 (Fig. 3E) exhibited limited growth inhibition in the liquid cultivation containing certain gel extracts but failed to form colonies on the corresponding solid medium (Table 1). This discrepancy suggests that the inability of microorganisms to form colonies on conventional solid medium is influenced by multiple factors.

### Toxicity of gelling agents was generated during autoclave

Because autoclaving is a standard step for the preparation of conventional solid medium, we investigated whether the toxicity of gelling agents originates during this process.

**Figure 4:**
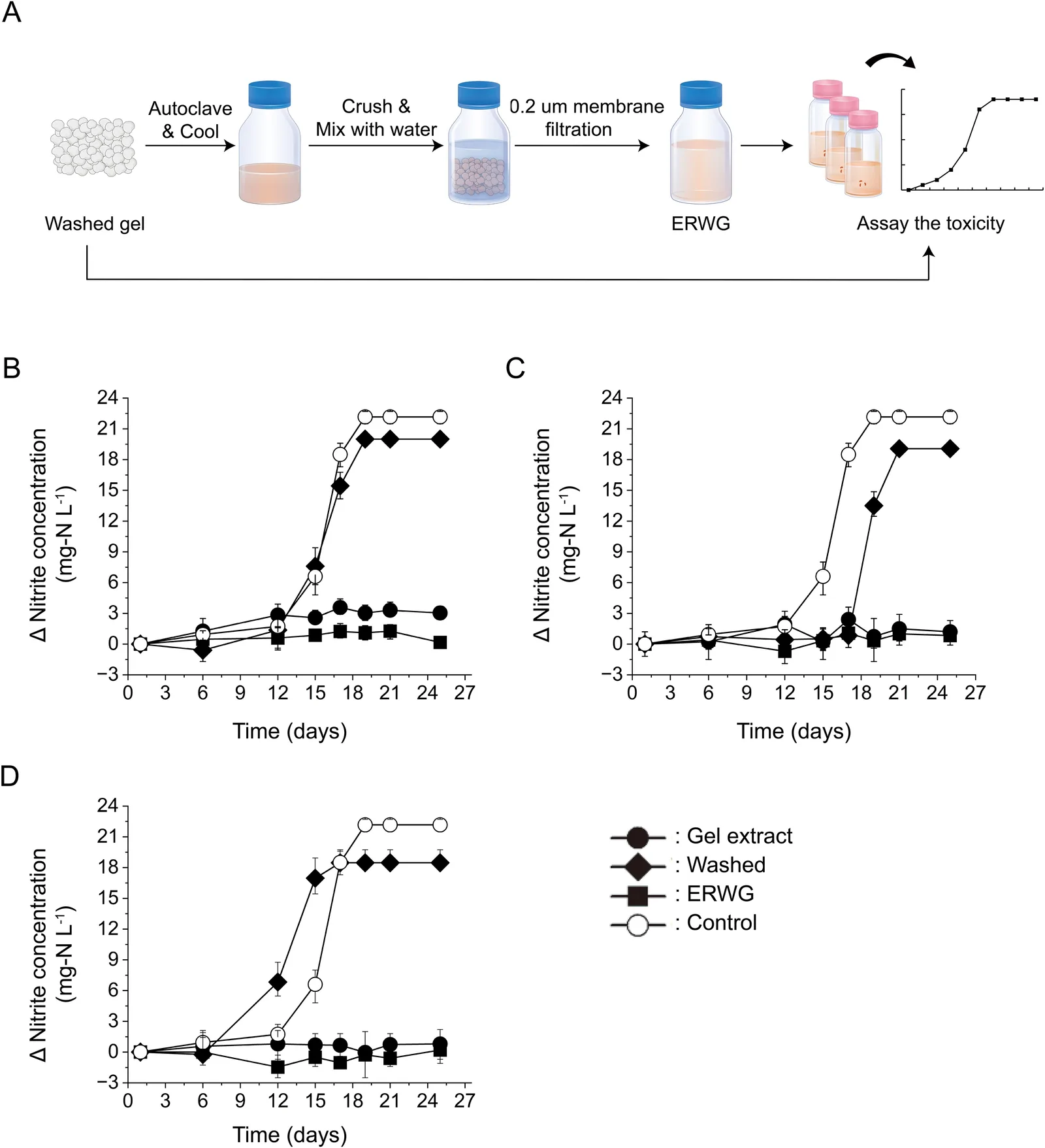
Effect of gel extract and ERWG to the growth of *Nitrospira* sp. ND1. (A) Experimental conception of making ERWG and comparing the toxicity of ERWG with washed gel and gel extract. (B–D) Growth of *Nitrospira* sp. ND1 in liquid medium with gel extract and ERWG derived from agar (B), agarose (C), and gellan gum (D). *X*-axis, incubation time (days); *Y*-axis, the changes in nitrite concentration (ΔNO₂^−^, mg-N L^−1^). Data represent means ± SD (*n* = 3).

The toxicity of washed gel was first tested by *Nitrospira* sp. ND1 to verify whether the toxic substances within gelling agents were successfully removed by the extraction process. Subsequently, the washed gel was re-autoclaved, and the extraction procedure was repeated two times to obtain the ERWG. The growth inhibitory effect of ERWG on *Nitrospira* sp. ND1 was then tested to compare its toxicity with that of the washed gel (Fig. 4A).

The washing step proved effective: no growth inhibition was detected in washed agar (Fig. 4B) or gellan gum (Fig. 4D), and only minimal inhibition occurred with washed agarose (Fig. 4C). In contrast, ERWGs strongly inhibited the growth of *Nitrospira* sp. ND1, indicating that the toxic substances within conventional solid medium were generated by autoclaving gelling agents, regardless of the gel types. These results indicate that inhibitory substances are generated during autoclaving of gelling agents.

### Growth of test strains on washed solid medium

**Figure 5:**
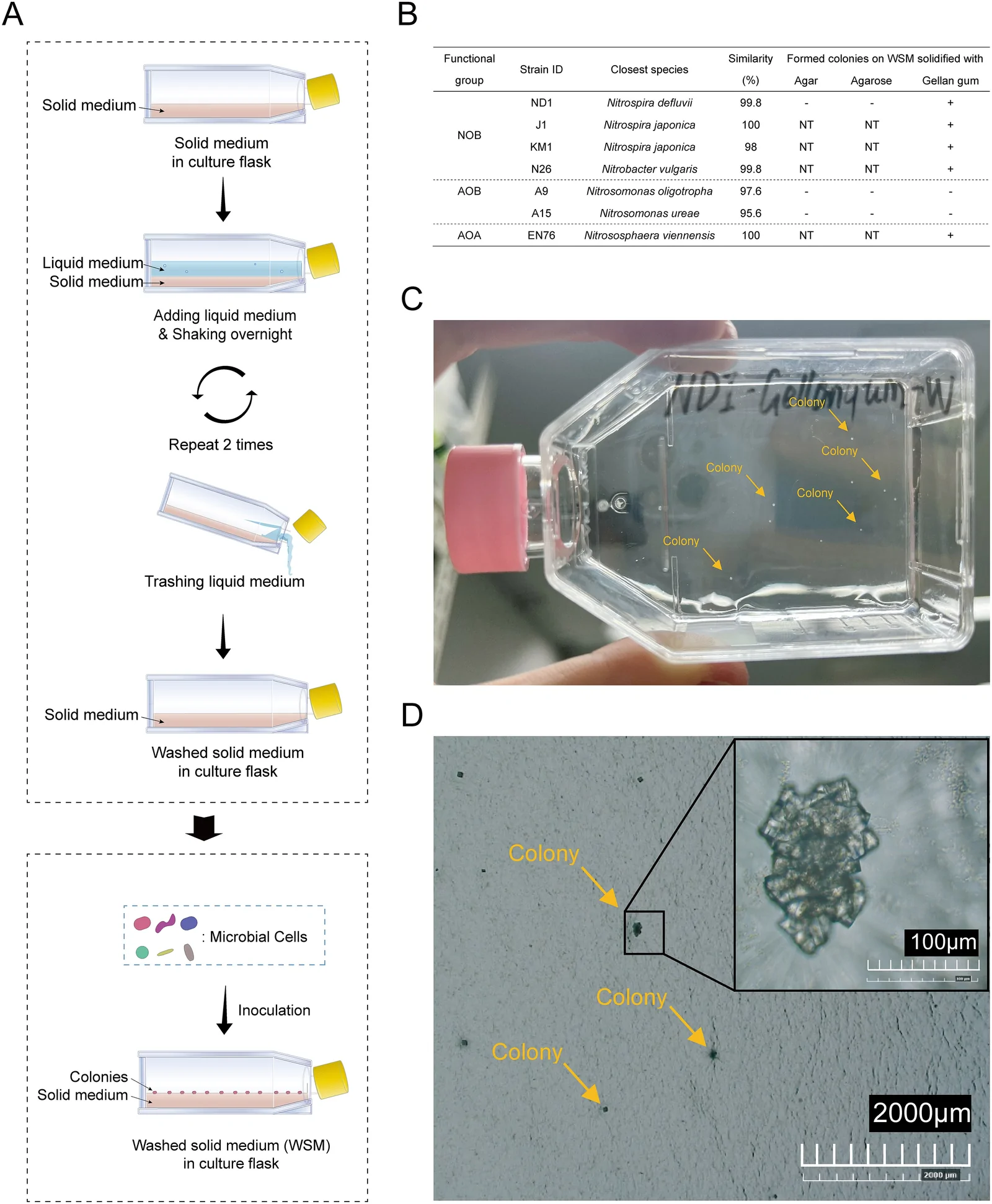
Colony formation of nitrifying strains on gellan gum-based washed solid medium. (A) The protocol of making washed solid medium and the experimental conception of cultivation microorganisms using washed solid medium. (B) Compare the colony formation ability of nitrifiers on gellan gum-based conventional solid medium and washed solid medium. “+” indicates that discrete colonies were observed, whereas “–” indicates no colony formation. (C) Macroscopic view of colonies of strain ND1 on washed solid medium, with representative colonies marked by yellow arrows. (D) Colonies of the strain EN76 on washed solid medium. The big panel is a low-magnification view (scale bar, 2000 μm) highlighting multiple colonies (yellow arrows), and the small panel is a higher-magnification microscopic image of a single colony from the boxed area (scale bar, 100 μm).

Based on these observations, we developed a simple washing strategy to create washed solid medium. We then tested whether this approach could enable colony formation for nitrifiers that failed to grow on conventional solid medium (Fig. 5A).

*Nitrospira* sp. ND1 formed colonies on gellan gum-based washed solid medium, whereas no colonies appeared on agar- or agarose-based washed solid medium (Fig. 5C). All tested NOB strains formed visible colonies on gellan gum-based washed solid medium. The AOA strain *N. viennensis* EN76 also produced visible colonies, reaching a size (~140 μm in length) sufficient for subculturing (Fig. 5D). Subsequent transfer of these colonies to liquid medium resulted in active nitrification, confirming their viability and identity.

In contrast, two AOB strains did not form colonies on washed solid medium regardless of the type of gelling agents (Fig. 5B).

To validate the applicability of this method beyond the nitrifying lineage, we applied washed solid medium to three heterotrophic test strains. We successfully cultivated strains No.120 and No.206, both of which had previously failed to grow on any type of conventional solid medium. Strain No.206 formed colonies on washed solid medium prepared with all three gelling agents (Fig. S2).

The successful recovery of colonies from all tested NOB strains on washed gellan gum medium, together with the colony formation of the AOA strain *N. viennensis* EN76 and three heterotrophic strains, demonstrates the effectiveness of washed solid medium in improving the culturability of diverse fastidious microorganisms.

*Nitrospira* sp. ND1 formed colonies only on washed gellan gum medium, whereas the two tested AOB strains failed to form colonies on any washed medium, indicating that colony formation on solid media is governed by multiple interacting factors and that the contribution of gelling agent**–**derived inhibitory compounds may differ among microorganisms and gelling agents. Although removal of gelling agent**–**derived inhibitory compounds alone is insufficient to overcome all cultivation barriers, it still represents an effective strategy for improving cultivability of microorganisms whose growth is primarily constrained by gelling agent–derived inhibition.

### Isolation of environmental nitrifiers using washed solid medium

**Figure 6:**
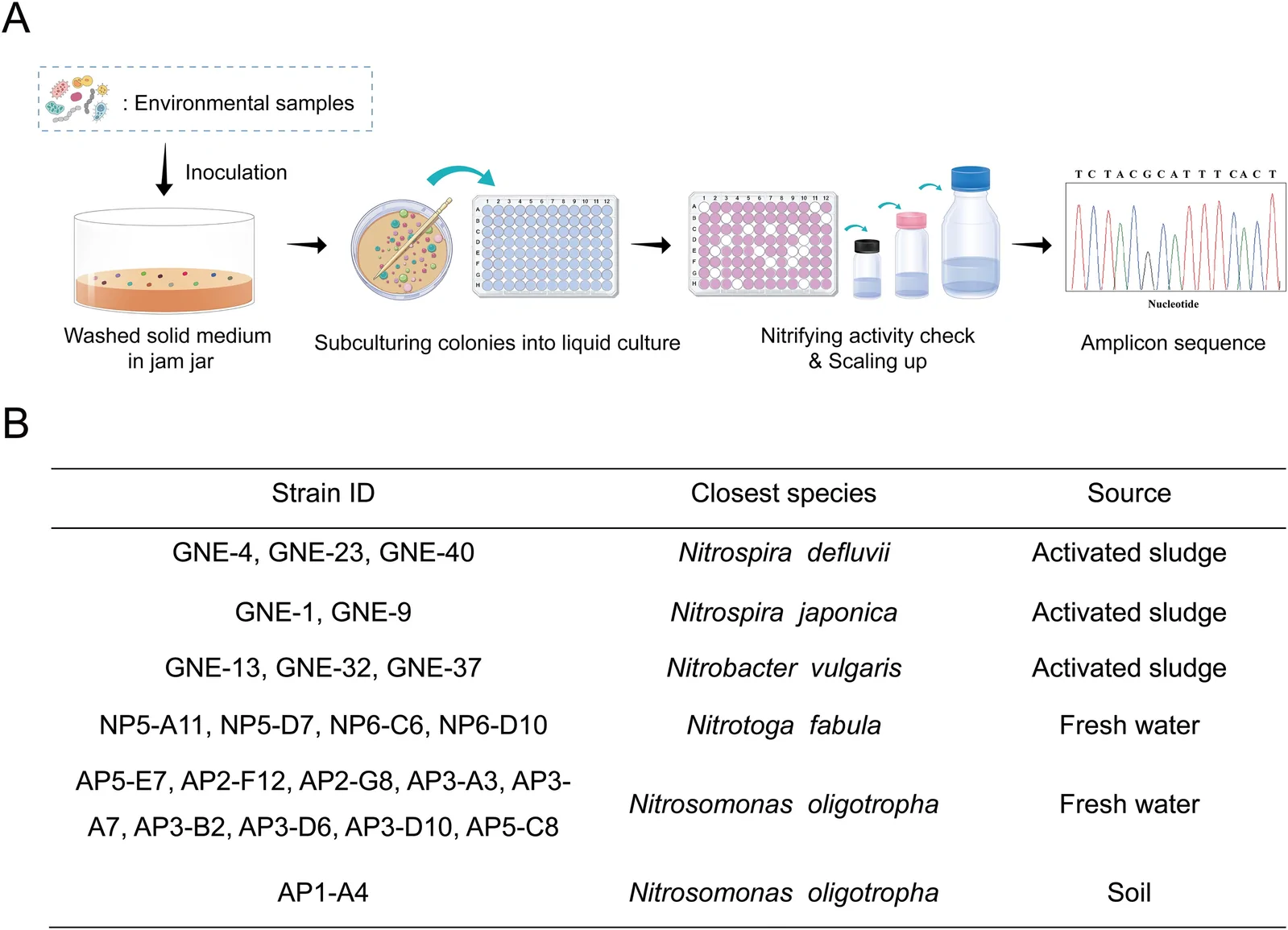
Cultivation of environmental nitrifiers using gellan gum-based washed solid medium. (A) Experimental conception of isolating environmental nitrifiers using gellan gum-based washed solid medium (B) The summarize of identified nitrifying colonies obtained from gellan gum-based washed solid medium. “Closest species” indicates the nearest described nitrifying species based on 16S rRNA gene sequence similarity. “Source” denotes the origin of the corresponding enrichment culture used for inoculation (AS, activated sludge enrichment culture; FW, fresh water enrichment culture; S, soil enrichment culture).

To further investigate whether the washed solid medium could be applied to the recovery of nitrifiers from complex microbial communities rather than from pure cultures alone, we employed gellan gum-based washed solid medium in isolating environmental nitrifiers from three enrichment cultures derived from activated sludge, soil, and freshwater (Fig. 6A).

As a result, the colonies of diverse nitrifiers were successfully obtained, including *Nitrosomonas, Nitrobacter, Nitrospira*, and *Nitrotoga*-like strains. In detail, 10 colonies related to *Nitrosomonas* were obtained from the enrichment cultures derived from soil and freshwater; 5 colonies related to *Nitrospira* and 3 colonies related to *Nitrobacter* were obtained from the activated sludge enrichment culture; and 1 colony related to *Nitrotoga* was obtained from the freshwater enrichment (Fig. 6B).

*Nitrospira* are the most abundant NOB in nature, natural and engineered systems. However, due to their notorious uncultivability, only 9 of the 158 detected *Nitrospira* species (5.6%) (Table S1) have been successfully cultivated. Members of the genus *Nitrotoga* have been detected across diverse habitats [76], yet only two species have been isolated, one from activated sludge and the other from a marine-related environment [29, 77]. The *Nitrotoga*-like strain obtained in this research represented the third isolate under *Nitrotoga* genus, and the first from a freshwater habitat. Therefore, the successful obtaining of multiple *Nitrospira* strains and one *Nitrotoga*-like strain in this study highlights the utility of washed solid medium for recovering fastidious nitrifiers.

Liquid cultivation methods, particularly dilution-to-extinction, remain the dominant approach for isolating environmental nitrifiers but are inherently limited by low throughput and the risk of heterotrophic contamination. These constraints reduce the likelihood of recovering low-abundance or rare taxa.

Solid medium offers several advantages for isolation of nitrifiers, but its application has been constrained by the growth inhibition associated with the toxicity of gelling agents. The washed solid medium developed in this study reduces this limitation, providing a practical, simple, scalable approach for isolation of environmental microorganisms.

## Conclusion

In this study, we show that commonly used gelling agents (agar, agarose, and gellan gum) generate water-soluble inhibitory substances upon autoclaving, which impair colony formation across diverse microbial taxa. These findings indicate that the limited culturability of microorganisms especially for nitrifiers on solid medium is not simply a property of the microorganisms themselves, but is strongly influenced by inhibitory effects associated with gelling agents.

To overcome this limitation, we developed washed solid medium that reduces these inhibitory factors. This method enabled the cultivation of previously recalcitrant microorganisms, such as several *Nitrospira* lineages and an AOA strain affiliated to *Nitrososphaera*. Moreover, this method enabled the successfully obtaining of environmental nitrifiers, including multiple strains of *Nitrospira, Nitrobacter*, and *Nitrosomonas*, as well as a *Nitrotoga* strain from a freshwater habitat. Although this approach may not support colony formation by every AOB strain, likely reflecting physiological diversity in their sensitivity to chemical stresses and local conditions on solid media, the successful isolation of *Nitrosomonas*-related AOB highlights the practical effectiveness of washed solid media for cultivating some AOB lineages.

Although the chemical identity of the inhibitory substances has not been resolved, our results establish their functional relevance and demonstrate that their removal substantially improves culturability. Future studies aimed at identifying these factors and elucidating how they suppress the growth of nitrifying microorganisms will further advance our understanding of microbial cultivation constraints.

## Supplementary Material

Supplementary_material_ycag246

## Data Availability

Sequence data and strain information have been deposited in the National Center for Biotechnology Information (GenBank: PZ177700–PZ177710) and the DNA Data Bank of Japan (BioProject accession: PRJDB40709).
