# Supplementary material for: Overcoming gelling agent–derived inhibitory factors enables the cultivation of fastidious nitrifiers on solid media
Source: ISME Commun. 2026 Sep 6;6(1):ycag246. doi: 10.1093/ismeco/ycag246 (PMC13584200; doi:10.1093/ismeco/ycag246)
Supplement: Supplementary file 1 [file supplementary_material_ycag246.zip › supplimentally table 1_ycag246.docx]

| Functional group | Family | Genus | Species |
| --- | --- | --- | --- |
| NOB (Including Comammox) | 8/11 | 11/41 | 20/215 |
| AOB | 1/1 | 3/7 | 22/108 |
| AOA | 2/17 | 4/74 | 12/619 |
| Total | 11/29 | 18/122 | 54/942 |
