# Supplementary material for: Overcoming gelling agent–derived inhibitory factors enables the cultivation of fastidious nitrifiers on solid media
Source: ISME Commun. 2026 Sep 6;6(1):ycag246. doi: 10.1093/ismeco/ycag246 (PMC13584200; doi:10.1093/ismeco/ycag246)
Supplement: Supplementary file 1 [file supplementary_material_ycag246.zip › supplimentally table 2_ycag246.docx]

| Functional group | Species | Isolation strategy | Source | Reference |
| --- | --- | --- | --- | --- |
| NOB | *Nitrospira defluvii* | Liquid method | Activated sludge | [55] |
|  | *Nitrospira marina* | Liquid method | Marine | / |
|  | *Nitrospira japonica* | Liquid method | Activated sludge | [18] |
|  | *Nitrospira moscoviensis* | Liquid method | Corroded area of an iron pipe | [22] |
|  | *Nitrospira tepida* | Liquid method | Activated sludge | [23] |
|  | *Nitrospira lenta* | Liquid method | Activated sludge | [53] |
|  | *Nitrospira calida* | Liquid method | Geothermal hot springs. | [35] |
|  | *Ca.* Nitrospira bockiana | Liquid method | Corroded area of an iron pipe | [50] |
|  | *Ca.* Nitrospira neomarina | Liquid method | Water column | [31] |
|  | *Nitrobacter winogradskyi* | Liquid method | / | [56] |
|  | *Nitrobacter hamburgensis* | Liquid method | Activated sludge | [38] |
|  | *Nitrobacter vulgaris* | Liquid method | Soil | [20] |
|  | *Nitrobacter alkalicus* | Liquid method | Alkaline soda lakes | [36] |
|  | *Nitrobacter agilis* | Liquid method | Soil | [34] |
|  | *Nitrococcus mobilis* | Liquid method | Marine | [27] |
|  | *Nitrospina gracilis* | Liquid method | Marine | [27] |
|  | *Nitrospina watsonii* | Liquid method | Marine | [28] |
|  | *Nitrotoga fabula* | Liquid method | Activated sludge | [29] |
|  | *Nitrolancea hollandica* | Liquid method | Activated sludge | [39] |
| Comammox | *Ca. Nitrospira inopinata* | Liquid method | biofilter | [11] |
| AOB | *Nitrosomonas cryotolerans* | Liquid method | Sea water | [21] |
|  | *Nitrosomonas supralitoralis* | Liquid method | Beach sand | [44] |
|  | *Nitrosospira briensis* | Liquid method | Soil | [51] |
|  | *Nitrosospira lacus* | Liquid method | Lake sediment | [49] |
|  | *Nitrosospira multiformis* | Liquid method | Soil | [19] |
|  | *Nitrosococcus oceani* | Liquid method | Sea water | [26] |
|  | *Nitrosococcus watsonii* | Liquid method | Marine sediment | [42] |
|  | *Nitrosococcus wardiae* | Solid method | Surface sediment | [32] |
|  | *Nitrosomonas europaea* | Solid method | Soil | [54] |
|  | *Nitrosomonas mobilis* | Solid method | Sea water | [37] |
|  | *Nitrosomonas stercoris* | Solid method | Cattle manure | [43] |
|  | *Nitrosospira tenuis* | Solid method | Soil | [24] |
|  | *Nitrosomonas aestuarii* | / | Sea water | [30] |
|  | *Nitrosomonas communis* | / | Soil | [30] |
|  | *Nitrosomonas eutropha* | / | Municipal sewage | [30] |
|  | *Nitrosomonas halophila* | / | Sea water | [30] |
|  | *Nitrosomonas marina* | / | Sea water | [30] |
|  | *Nitrosomonas nitrosa* | / | Pond | [30] |
|  | *Nitrosomonas oligotropha* | / | Soil | [30] |
|  | *Nitrosomonas ureae* | / | Soil | [30] |
|  | *Nitrosococcus nitrosus* | / | / | / |
|  | *Nitrosococcus halophilus* | / | Salt lake | [33] |
|  | *Nitrosotalea sinensis* | Liquid method | Soil | [25] |
|  | *Nitrosotalea devaniterrae* | Liquid method | Soil | [52] |
|  | *Nitrososphaera viennensis* | Liquid method | Soil | [48] |
|  | *Nitrosopumilus zosterae* | Liquid method | Sea water | [43,47] |
|  | *Nitrosopumilus ureiphilus* | Liquid method | Surface sediment | [46] |
|  | *Nitrosopumilus oxyclinae* | Liquid method | Sea water | [46] |
|  | *Nitrosopumilus cobalaminigenes* | Liquid method | Sea water | [46] |
| AOA | *Nitrosopumilus piranensis* | Liquid method | Sea water | [45] |
|  | *Nitrosopumilus adriaticus* | Liquid method | Sea water | [45] |
|  | *Nitrosopumilus maritimus* | Liquid method | Sea water | [12] |
|  | *Nitrosoarchaeum haohaiensis* | Liquid method | Sea water | [40] |
|  | *Nitrosarchaeum koreense* | Liquid method | Soil | [41] |
