# Supplementary material for: Overcoming gelling agent–derived inhibitory factors enables the cultivation of fastidious nitrifiers on solid media
Source: ISME Commun. 2026 Sep 6;6(1):ycag246. doi: 10.1093/ismeco/ycag246 (PMC13584200; doi:10.1093/ismeco/ycag246)
Supplement: Supplementary file 1 [file supplementary_material_ycag246.zip › supplimentally table 3_ycag246.docx]

| Functional group | Strain ID | Closest species | Similarity  (%) | Solid media for colony formation assay | Substances for  growth inhibition assay | Reference |
| --- | --- | --- | --- | --- | --- | --- |
| NOB | NDI | *Nitrospira defluvii* | 99.8 | CSM, WSM, LS | GE, C+GE,WG,  ERWG, CaCl_2_ | [55] |
|  | J1 | *Nitrospira japonica* | ※ | CSM, WSM | GE | [18] |
|  | KM1 | *Nitrospira japonica* | 98.0 | CSM, WSM | GE | [58] |
|  | N26 | *Nitrobacter vulgaris* | 99.8 | CSM, WSM | GE | This study |
| AOB | A9 | *Nitrosomonas oligotropha* | 97.6 | CSM, WSM | GE | This study |
|  | A15 | *Nitrosomonas ureae* | 95.6 | CSM, WSM | GE | This study |
| AOA | EN76 | *Nitrososphaera viennensis* | ※ | CSM, WSM | GE | [48] |
|  | NF5 | *Nitrosopumilus adriaticus* | ※ | CSM, WSM | GE | [45] |
| Heterotrophs | No.120 | *Rhizorhabdus wittichii* | 96.0 | CSM, WSM | GE | [59] |
|  | No.154 | *Novosphingobium pentaromativorans* | 97.0 | CSM, WSM | GE | [59] |
|  | No.206 | *Reyranella massiliensis* | 99.0 | CSM, WSM | GE | [59] |
|  | No.1 | *Escherichia coli* | ※ | CSM | GE | [80]  This study  This study |
|  | No.62 | *Nocardioides pyridinolyticus* | 97.0 | CSM | GE |  |
|  | No.508 | *Skermanella aerolata* | 92.4 | CSM | GE |  |
