# Supplementary material for: Overcoming gelling agent–derived inhibitory factors enables the cultivation of fastidious nitrifiers on solid media
Source: ISME Commun. 2026 Sep 6;6(1):ycag246. doi: 10.1093/ismeco/ycag246 (PMC13584200; doi:10.1093/ismeco/ycag246)
Supplement: Supplementary file 1 [file supplementary_material_ycag246.zip › supplimentally table 4_ycag246.docx]

| Functional  Group | Strain ID | Closest species | Similarity  (%) | Growth on CSM solidified with | | | Growth in liquid medium with extract of | | |
| --- | --- | --- | --- | --- | --- | --- | --- | --- | --- |
|  |  |  |  | Agar | Agarose | Gellan gum | Agar | Agarose | Gellan gum |
| Heterotrophs | No.120 | *Sphingomonas wittichii* | 96.0 | **-** | **-** | **-** | **-** | **-** | **-** |
|  | No.154 | *Novosphingobium pentaromativorans* | 97.0 | **-** | **-** | + | **-** | **-** | + |
|  | No.206 | *Reyranella massiliensis* | 99.0 | **-** | **-** | **-** | **-** | **-** | **-** |
